# Motion correction of free-breathing magnetic resonance renography using model-driven registration

**DOI:** 10.1007/s10334-021-00936-x

**Published:** 2021-06-23

**Authors:** Dimitra Flouri, Daniel Lesnic, Constantina Chrysochou, Jehill Parikh, Peter Thelwall, Neil Sheerin, Philip A. Kalra, David L. Buckley, Steven P. Sourbron

**Affiliations:** 1 Department of Applied Mathematics, University of Leeds, Leeds, UK; 2 Department of Biomedical Imaging Sciences, University of Leeds, Leeds, UK; 3 School of Biomedical Engineering and Imaging Sciences, Kings College London, London, UK; 4 Department of Medical Physics and Biomedical Engineering, University College London, London, UK; 5 Department of Renal Medicine, Salford Royal National Health Service Foundation Trust, Salford, UK; 6 Translational and Clinical Research Institute, Newcastle University, Newcastle upon Tyne, UK; 7 Newcastle Magnetic Resonance Centre, Campus for Ageing and Vitality, University of Newcastle, Newcastle upon Tyne, UK; 8 Department of Infection, Immunity and Cardiovascular Disease, University of Sheffield, Sheffield, UK

**Keywords:** Model-driven registration, Motion correction, Image registration, Free-form deformation, Quantitative imaging, Magnetic resonance renography, Dynamic contrast-enhanced MRI

## Abstract

**Introduction:**

Model-driven registration (MDR) is a general approach to remove patient motion in quantitative imaging. In this study, we investigate whether MDR can effectively correct the motion in free-breathing MR renography (MRR).

**Materials and methods:**

MDR was generalised to linear tracer-kinetic models and implemented using 2D or 3D free-form deformations (FFD) with multi-resolution and gradient descent optimization. MDR was evaluated using a kidney-mimicking digital reference object (DRO) and free-breathing patient data acquired at high temporal resolution in multi-slice 2D (5 patients) and 3D acquisitions (8 patients). Registration accuracy was assessed using comparison to ground truth DRO, calculating the Hausdorff distance (HD) between ground truth masks with segmentations and visual evaluation of dynamic images, signal-time courses and parametric maps (all data).

**Results:**

DRO data showed that the bias and precision of parameter maps after MDR are indistinguishable from motion-free data. MDR led to reduction in HD (HD_unregistered_ = 9.98 ± 9.76, HD_registered_ = 1.63 ± 0.49). Visual inspection showed that MDR effectively removed motion effects in the dynamic data, leading to a clear improvement in anatomical delineation on parametric maps and a reduction in motion-induced oscillations on signal-time courses.

**Discussion:**

MDR provides effective motion correction of MRR in synthetic and patient data. Future work is needed to compare the performance against other more established methods.

## Introduction

Quantitative imaging biomarkers are measured by acquiring images with different acquisition parameters, and fitting a physical model of the ensuing signal changes to these data. For body areas like the abdomen and thorax, motion correction is often necessary to avoid significant artefacts in the parametric maps. Free-breathing magnetic resonance renography (MRR) [1], or dynamic contrast-enhanced MRI (DCE-MRI) of the kidney, exhibits a combination of characteristics that present extremely challenging conditions for motion correction. This includes major reversals in image contrast and large motion amplitudes compared to the internal structures of the target organ. As a result, and despite intensive research in the area, a sufficiently robust motion-correction approach for MRR has not yet been identified [2–6].

Standard pairwise image co-registrations between the images in the series [7, 8] or with a single reference image is often ineffective in quantitative imaging due to the large changes in image contrast. The problem can be solved by adopting a more group wise perspective and considering the motion correction of all images in the series as a joint optimization problem. Two different classes of methods can be distinguished: (1) Model-driven registration (MDR) methods generate target images by fitting the physical signal model to the data [9] and perform a (usually pairwise) co-registration between source images and targets. (2) Model-free registration methods do not rely on the physical signal model and instead use more general signal-processing concepts to provide a cost function for the groupwise co-registration problem [10–17].

The attraction of model-free registration lies in the fact that the same algorithm can be applied to any quantitative imaging method, and the results are not affected by biases in the physical signal model [18]. On the other hand, motion correction is not an aim in itself in quantitative imaging, but part of a processing pipeline that will ultimately always apply a signal model. Hence in this context, model-free registration can impose additional constraints that may bias the solution unnecessarily. For instance, the assumption that breathing does not affect the principal components of a signal is not necessarily true. In particular, this may be the case when data are acquired rapidly in free-breathing and motion creates a large periodic oscillation on the signal.

MDR does not impose additional assumptions beyond those implicit in the physical signal model, and may therefore be a more suitable motion-correction approach in quantitative imaging [19]. Evidence shows that MDR performs well in a wide range of application areas, but the majority of these involve data with relatively small amounts of motion, such as breast [9] and brain [20–22] (immobilised by dedicated coils), ECG-triggered cardiac or abdominal data in breath hold [23–29] or lower abdomen [30–32] (limited bulk motion and peristalsis suppressed with antispasmodics [33]). Only two studies so far have applied MDR in free-breathing [19, 34], both on abdominal diffusion-weighted MRI which does not exhibit the large reversals in contrast shown by T1-mapping or DCE-MRI. MDR has not yet been evaluated as a motion correction method for MRR.

The aim of this study was to investigate whether MDR is a suitable approach to correct for breathing motion in high temporal resolution MRR acquired under free-breathing. The standard formulation of MDR was generalised to cover this type of problem, and MDR was evaluated on a kidney-mimicking digital reference object (DRO), as well as on patient data using multi-slice 2D and 3D acquisitions. The algorithm, DRO and patient data are freely available as supplementary material to allow independent verification of the conclusions and serve as benchmarks for future developments in motion correction (github.com/plaresmedima/Flouri et al. 2020).

## Materials and methods

### Theory

This section introduces basic theory and notations, and generalises conventional MDR for application to MRR with linearised kinetic models. The theory is introduced in the most general context possible to allow transfer of experience to other applications of quantitative imaging and DCE-MRI.

#### DCE-MRI signal model

The basic approach to modelling of MRR is well-known [1] and follows standard principles of DCE-MRI, but some of the usual distinctions are irrelevant for the purposes of motion correction. We repeat here the main definitions to unambiguously fix notations and assumptions.

DCE-MRI measures $S\left( {\user2{x},t} \right)$ in location ***x*** as a function of time *t* after injection of a contrast agent. For the purposes of this study, we will assume that tissue concentrations are small enough so that $S\left( {\user2{x},t} \right)$ is approximately linear in the concentration [35]:

1 $$S\left( {\user2{x},t} \right) = S_{0} \left( \user2{x} \right) + \rho \left( \user2{x} \right)C\left( {\user2{x},t} \right)$$

Here, $\rho \left( \user2{x} \right) = r_{1} S_{0} \left( \user2{x} \right)T_{{10}} \left( \user2{x} \right)$ depends on the relaxivity $r_{1}$ of the contrast agent, the pre-contrast relaxation time $T_{{10}}$ and on the pre-contrast signal $S_{0}$. We will also assume that the tracer distributes over at most two distinct compartments with a global input function $c_{a} \left( t \right)$. In that case, $C\left( {\user2{x},t} \right)$ is a convolution of $c_{a} \left( t \right)$ with a bi-exponential:

2 $$C\left( {\user2{x},t} \right) = \mathop \sum \limits_{{i = 1}}^{2} F_{i} \left( \user2{x} \right)e^{{ - t/T_{i} \left( {\mathbf{x}} \right)}} \otimes c_{a} \left( t \right)$$

Here, $F_{i} \left( \user2{x} \right)$ and $T_{i} \left( \user2{x} \right)$ are four unknown parameter fields. The model is easily generalised to more complex systems by adding terms in the sum, one for each compartment [21, 36]. Inserting Eq. (2) into Eq. (1) provides the main model equation for two-compartmental DCE-MRI:

3 $$S\left( {\user2{x},~t} \right) = S_{0} \left( \user2{x} \right) + \mathop \sum \limits_{{i = 1}}^{2} F_{i} \left( \user2{x} \right)e^{{ - t/T_{i} \left( {\mathbf{x}} \right)}} \otimes c_{a} \left( t \right)$$

Here, we absorbed the calibration map $\rho \left( \user2{x} \right)$ into a re-definition $F_{i} \to \rho F_{i}$, as the parameter interpretation is not relevant for motion correction purposes.

To avoid a dependence on initial values and speed up model fitting, a linear formulation of the two-compartment model can also be used [37]:

4 $$C\left( {\user2{x},t} \right) = \mathop \sum \limits_{{i = 1}}^{2} \left( {\alpha _{i} \left( \user2{x} \right)C^{{\left( i \right)}} \left( {\user2{x},t} \right) + \beta _{i} \left( \user2{x} \right)c_{a}^{{\left( i \right)}} \left( t \right)} \right)$$

Here, the superscripts ‘(1)’ and ‘(2)’ refer to single and double integration over time, respectively. The unknown model parameters are the four fields $\alpha _{i} \left( \user2{x} \right)$ and $\beta _{i} \left( \user2{x} \right)$, which are directly related to the four parameters $F_{i} \left( \user2{x} \right)$ and $T_{i} \left( \user2{x} \right)$ [37]. Inserting Eq. (4) into Eq. (1) leads to the signal model for linear DCE-MRI:

5 $$S\left( {\user2{x},~t} \right) = S_{0} \left( \user2{x} \right) + \mathop \sum \limits_{{i = 2}}^{2} \left( {\alpha _{i} \left( \user2{x} \right)\left( {S(\user2{x},t} \right) - S_{0} \left( \user2{x} \right))^{{\left( \user2{i} \right)}} + \beta _{i} \left( \user2{x} \right)c_{a}^{{\left( i \right)}} \left( t \right)} \right)$$

As above we absorbed $\rho \left( \user2{x} \right)$ into a re-definition $\beta _{i} \to \rho \beta _{i}$.

#### Free-form deformation model (FFD)

Consider a set of images $S\left( {\user2{x},~t} \right)$ acquired at different times *t* and corrupted by motion. If motion is modelled by a coordinate transformation $\user2{x} \to \user2{D}\left( {\user2{x},~t} \right)$ at each time point $t$ (the deformation field at $t$), then motion-free images $S_{\user2{D}} \left( {\user2{x},~t} \right)$ can be derived as:

6 $$S_{\user2{D}} \left( {\user2{x},~t} \right) = S\left( {\user2{D}\left( {\user2{x},~t} \right),t} \right)$$

We are free to define which is considered the motion-free state $\user2{D}\left( {\user2{x},~t_{f} } \right) = \user2{x}$. In a free-breathing protocol, it is convenient to choose ${t}_{f}$ in end-expiration as this typically covers the largest part of a breathing cycle.

The breathing motion in this study is modelled with free-form deformations (FFD) [7]. The deformation field $\user2{D}\left( {\user2{x},~t} \right)$ is defined at any ${\mathbf{x}}$ by interpolating vectors $\user2{D}_{j} \left( t \right) = \user2{D}\left( {\user2{x}_{j} ,t} \right)$ on a rectangular grid of control points $\user2{x}_{j}$ with grid spacing $\Delta = \left( {\Delta _{x} ,\Delta _{y} ,\Delta _{z} } \right)$:

7 $$\user2{D}\left( {\user2{x},~t} \right) = \mathop \sum \limits_{j} W_{j}^{\Delta } \left( \user2{x} \right)\user2{D}_{j} \left( t \right)$$

In this study, we use linear interpolation to speed up the computations, in which case the weighting functions $W_{j}^{\Delta } \left( \user2{x} \right) = W^{\Delta } (\user2{x} - \user2{x}_{j} )$ are defined as:

8 $$W^{\Delta } \left( {x,y,z} \right) = w\left( {x/\Delta x} \right)w\left( {y/\Delta y} \right)w\left( {z/\Delta z} \right)$$

with $w\left( u \right) = 1 - \left| u \right|$ for $\left| u \right| \le 1$ and $w\left( u \right) = 0~$ otherwise. The coordinate transformation (Eq. 6) is applied by interpolating the images between the values $S_{l} \left( t \right) = S(\user2{x}_{l} ,t)~$ at voxel centres $\user2{x}_{l}$. If the voxel dimensions are *δ*, the signal at any location ${\mathbf{x}}$ is given by:

9 $$S_{\user2{D}} \left( {\user2{x},~t} \right) = \mathop \sum \limits_{l} W_{l}^{\delta } \left( \user2{x} \right)S_{l} \left( t \right)$$

The deformed image (Eq. 6) can then be parametrized in terms of $\user2{D}_{j} \left( t \right)$ by substituting $\user2{x} \to \user2{D}\left( {\user2{x},~t} \right)$ in the right-hand side of Eq. (9) and using Eq. (7):

10 $$S_{\user2{D}} \left( {\user2{x},~t} \right) = \mathop \sum \limits_{l} W_{l}^{\delta } \left( {\user2{D}\left( {\user2{x},~t} \right)} \right)S_{l} \left( t \right)$$

#### Standard MDR (fixed target)

Consider an arbitrary quantitative imaging method where signals $S\left( {\user2{x},~t} \right)$ are measured at locations ${\mathbf{x}}$ and at times $t$ with different signal parameters $\user2{p}\left( t \right)$. Depending on modality, ***p*** can represent time points directly (e.g., DCE-MRI or Dynamic PET), b-values and gradient directions (diffusion MRI), inversion or echo times (MRI relaxometry), phase-encoding directions (4D Flow), etc.

Standard MDR assumes there exists a signal model $\sum {\left( {\user2{P}\left( \user2{x} \right),\user2{p}} \right)}$ that describes motion-free images in terms of $N$ unknown parameter maps $\user2{P}\left( \user2{x} \right) = \left( {P_{1} \left( \user2{x} \right), \ldots ,P_{N} \left( \user2{x} \right)} \right)$:

11 $$S_{\user2{D}} \left( {\user2{x},~t} \right) = \Sigma \left( {\user2{P}\left( \user2{x} \right),\user2{p}\left( t \right)} \right)$$

The left-hand side is here the motion-corrected image as defined in Eq. (6). Equation (11) is the defining equation of standard MDR, providing a direct link between deformation fields $\user2{D}\left( {\user2{x},~t} \right)$ and parameter maps $\user2{P}\left( \user2{x} \right)$.

Equation 11 is solved by initialising $\user2{D}\left( {\user2{x},~t} \right) = \user2{x}$ and iterating the following two steps until convergence: (1) solve for ***P*** at fixed ***D***; (2) solve for ***D*** at fixed ***P***. This breaks up the problem in a series of smaller problems that can be addressed with existing methods: solving for ***P*** at fixed ***D*** amounts to fitting the signal model to a series of images $S_{\user2{D}} \left( {\user2{x},~t} \right)$; solving for ***D*** at fixed ***P*** presents a standard pairwise image co-registration problem for each *t* that can be solved with common software packages.

While image co-registration generally requires normalised cost functions to account for differences in contrast and intensity between source and target, in MDR, a least squares cost function can be used because the source images $S_{\user2{D}} \left( {\user2{x},~t} \right)$ and target images $\Sigma \left( {\user2{P}\left( \user2{x} \right),\user2{p}\left( t \right)} \right)$ have similar intensity and contrast. Since the physical signal models are typically also solved in the least squares sense, this allows us to formulate MDR as a joint optimization problem:

12 $$\chi ^{2} \left( {\user2{D},\user2{P}} \right) = \frac{1}{2}\mathop \sum \limits_{{\user2{x},t}} \left( {S_{\user2{D}} \left( {\user2{x},t} \right) - \Sigma \left( {~\user2{P}\left( \user2{x} \right),\user2{p}\left( t \right)} \right)} \right)^{2}$$

Since both steps of the iteration then optimize the same cost function, convergence is guaranteed. The use of a least squares cost function also offers a significant computational advantage because it affords an analytical expression for the partial derivatives $\user2{G}_{j} \left( t \right)$ of the cost function with respect to each $\user2{D}_{j} \left( t \right)$ [38]:

13 $$\user2{G}_{j} \left( t \right) = \sum\limits_{{{\mathbf{x}} \in {\mathcal{N}}_{j} }} {W_{j}^{\Delta } } \left( \user2{x} \right)R\left( {\user2{x},t} \right)\left( {\nabla S} \right)_{\user2{D}} \left( {\user2{x},t} \right)$$

Here $\left( {\nabla S} \right)_{\user2{D}}$ is the FFD of the image gradient $\nabla S$, $R\left( {\user2{x},t} \right)$ is the residual $S_{\user2{D}} \left( {\user2{x},~t} \right) - \Sigma \left( {\user2{P}\left( \user2{x} \right),\user2{p}\left( t \right)} \right)$, and ${\mathcal{N}}_{j}$ is the support of the wavelet $W_{j}^{\Delta } \left( \user2{x} \right)$ (Eq. 8).

Equation (13) can be computed efficiently. The weights $W_{j} \left( \user2{x} \right)$ only depend on the resolution level Δ and can be pre-computed for each control point $j$. The product $R\left( {\user2{x},t} \right)~\left( {\nabla S} \right)_{\user2{D}} \left( {\user2{x},t} \right)$ needs to be computed only once for all control points $j$, and the summation over ***x*** can be restricted to a small neighbourhood ${\mathcal{N}}_{j}$ around each control point $j$ (Eq. 8).

#### Generalised MDR (moving target)

A hidden assumption in the formulation of standard MDR is that the signal model $\Sigma \left( {\user2{P}\left( \user2{x} \right),\user2{p}} \right)$ is not a function of the measured signals $S\left( {\user2{x},\user2{p}} \right)$ themselves. In general, however, this is not necessarily the case.

Consider the example of the DCE-MRI model in Eq. (3). If $S_{0} \left( \user2{x} \right)$ is treated as an unknown model parameter, then Eq. (3) defines a 5-parameter model $\Sigma (S_{0} \left( \user2{x} \right),F_{{\text{i}}} \left( \user2{x} \right),~T_{{\text{i}}} \left( \user2{x} \right),t)$, and the assumptions of standard MDR are fulfilled. In practice, however, nearly all current implementations of DCE-MRI derive $S_{0} \left( \user2{x} \right)$ from the data by averaging the pre-contrast signals $S\left( {\user2{x},~1} \right), \ldots ,S\left( {\user2{x},~n_{0} } \right)$. In that case, $\Sigma \left( {S\left( {\user2{x},1~:n_{0} } \right),F_{{\text{i}}} \left( \user2{x} \right),~T_{{\text{i}}} \left( \user2{x} \right),t} \right)$ becomes a function of the signal at different times. This is also true when a linear implementation is used as in Eq. (5), which depends explicitly on $S\left( {\user2{x},t} \right) =$ even if $S_{0} \left( \user2{x} \right)$ is treated as a free parameter.

The implications are significant. If the signal model depends on the signal itself, then Eq. (11) must be recast as follows^1^:

14 $$S_{\user2{D}} \left( {\user2{x},t} \right) = \Sigma \left( {S_{\user2{D}} \left( {\user2{x}, - } \right),~\user2{P}\left( \user2{x} \right),\user2{p}\left( t \right)} \right)$$

Solving this equation for $\boldsymbol{D}$ at fixed $\boldsymbol{P}$ no longer presents a standard image co-registration problem because the right-hand side also depends on $\boldsymbol{D}$—presenting effectively a moving target. An additional problem is that a deformation at any single time $t$ can now affect the equation at any other time $t'$. This implies that co-registrations at different times can no longer be treated as independent problems, and the problem requires a groupwise optimization of the cost function:

15 $$\chi ^{2} \left( {\user2{D},\user2{P}} \right) = \frac{1}{2}\mathop \sum \limits_{{\user2{x},t}} \left( {S_{\user2{D}} \left( {\user2{x},t} \right) - \Sigma \left( {S_{\user2{D}} \left( {\user2{x}, - } \right),~\user2{P}\left( \user2{x} \right),\user2{p}\left( t \right)} \right)} \right)^{2}$$

As shown in the appendix, the gradient of the cost function (Eq. 15) has the same form as the special case (Eq. 13), after substituting a generalised residual $R\left( {\user2{x},t} \right) \to {\mathcal{R}}\left( {\user2{x},t} \right)R$:

16 $${\mathcal{R}}\left( {\user2{x},s} \right) = \mathop \sum \limits_{s} R(\user2{x},s)~\left( {\partial _{t} R} \right)\left( {\user2{x},s} \right)$$

We used the notation $\left( {\partial _{t} R} \right)\left( {\user2{x},s} \right)$ for the partial derivative of the residual $R\left( {\user2{x},s} \right) = S_{D} \left( {\user2{x},s} \right) - \sum \left( {\user2{S}_{\user2{D}} \left( {\user2{x},s} \right)\left( {\user2{x}, - } \right),~\user2{P}\left( \user2{x} \right),\user2{p}\left( s \right)} \right)$ with respect to $S_{D} \left( {\user2{x},s} \right)$. In the special case of Eq. (12), we have $\left( {\partial _{t} R} \right)\left( {\user2{x},s} \right) = \delta _{{ts}}$ so that ${\mathcal{R}}\left( {\user2{x},s} \right) = R\left( {\user2{x},s} \right)$.

The need for a groupwise optimization in generalized MDR comes with a significant computational overhead. However, in the case of DCE-MRI with a linear model (Eq. 5), the assumption $\left( {\partial _{t} R} \right)\left( {\user2{x},s} \right) \approx \delta _{{ts}}$ may well present a reasonable approximation to the exact gradient. Defining a matrix $\partial \user2{R}\left( \user2{x} \right)$ with elements $\left( {\partial \user2{R}} \right)_{{ts}} \left( \user2{x} \right) = \left( {\partial _{t} R} \right)\left( {\user2{x},s} \right)$, and writing ***I*** for the identity matrix, $\Delta t\user2{{\rm M}S}$ for numerical integration of ***S*** over time, and $\user2{{\rm M}}_{0} \user2{S}$ for the K-element vector $\left( {S_{0} , \ldots ,S_{0} } \right)^{T}$, we can write the partial derivative of the residual as:

17 $$~\partial \user2{R}\left( \user2{x} \right) = (1 - \in _{1} \left( \user2{x} \right)\user2{{M}} - \in _{2}^{2} \left( \user2{x} \right)\user2{{M}}^{2} \left( {{\user2{I}} - \user2{{M}}_{0} } \right)$$

where we used the definition of $\alpha _{i}$ and $\beta _{i}$ [37] to define:

18 $$\in _{1} = \frac{{\Delta t}}{{T_{1} \left( \user2{x} \right)}} + \frac{{\Delta t}}{{T_{2} \left( \user2{x} \right)}}\, \in _{2} = \sqrt {\frac{{\Delta t}}{{T_{1} \left( \user2{x} \right)}}\frac{{\Delta t}}{{T_{2} \left( \user2{x} \right)}}}$$

In DCE-MRI, the time step ∆*t* is always chosen to be significantly shorter than the mean transit times of the system, so that $\Delta t \ll T_{1} ,~T_{2}$ and therefore $\in _{1} , \in _{2}$ are small quantities. Since further, the non-zero elements of the matrix $\user2{M}_{0}$ are 1*/*$n_{0}$ (with $n_{0}$ ∼ 10–20 the number of pre-contrast images), the approximation $\partial \user2{R} \approx \user2{I}$ may be justified. In that case, the dependency on ***D*** of the right-hand side in Eq. (14) can be ignored and the equation can be solved in the same way as standard MDR. This approach has been adopted in the current study.

### Implementation

MDR was implemented with the linear DCE-MRI signal model (Eq. 4). A multi-resolution strategy was adopted where the deformation fields $\user2{D}_{j} \left( t \right)$ are initially determined over a coarse grid of control points with spacing ∆ equal to the field of view. The solutions $\user2{D}_{j} \left( t \right)$ are then interpolated to initialise deformation vectors at a finer grid ∆ ⟶ ∆*/*2. This process is iterated until a user-defined minimum grid spacing ∆_min_. Deformation fields at the lowest resolution were initialised to no deformation: $\user2{D}_{j} \left( t \right) = \user2{x}_{j}$.

At each fixed resolution level ∆, the interpolation weights (Eq. 8) were pre-computed and the following two steps were iterated: (1) pixel-by-pixel linear least squares fitting of the four model parameters $\alpha _{i} \left( \user2{x} \right),~\beta _{i} \left( \user2{x} \right)$ to the deformed images $S_{\user2{D}} \left( {\user2{x},t} \right)$[37]; (2) time-by-time fitting of the deformation parameters $\user2{D}_{j} \left( t \right)$ using gradient descent with a back-tracking line search and gradients calculated with Eq. (13). The line searches were preconditioned by using the step size of the previous line search to initialise the next. The gradient descent was stopped if the line search returned a maximum change in $\user2{D}_{j} \left( t \right)$ less than a user-defined tolerance *δ*_min_. The iterations at a given resolution level were stopped if none of the time points produced a change larger than *δ*_min_.

Optimal values for the minimum grid spacing ∆_min_ and the tolerance *δ*_min_ were found for a test case for each type of data in two steps. First, the grid spacing ∆_min_ was varied at the lowest tolerance considered (*δ*_min_ = 0.1 pixels) and an optimal grid spacing was identified. Second, to minimize computation times, the tolerance *δ*_min_ was varied between 1.0 and 0.1 for the optimal grid spacing ∆_min_ selected in the previous step. The smallest tolerance beyond which no substantial improvement was apparent was identified.

MDR was implemented in IDL 6.4 (Exelis VIS, Boulder, CO) and run on a standard laptop PC with a 2.7 GHz Intel Core processor and 16 GB memory.

### Data and simulations

MDR was evaluated using a combination of 2D simulated data and patient data acquired in 2D and 3D. All patients provided written informed consent and the studies were approved by the local research ethics committees. The 3D data were co-registered with 3D MDR where $\user2{D}_{j} \left( t \right)$ has 3 independent components. 2D data were co-registered with 2D MDR.

#### Digital reference object (DRO)

A 2D DRO with kidney-like structures was used to generate motion-corrupted MRR images (120 dynamics, 1.1 s intervals, matrix size 135 × 135). The “kidneys” were modelled as concentric ellipses with different values for the parameters ${F}_{P}$ and ${T}_{T}$ to model cortical, medullary, and pelvic structures. The parameter values in the different regions range from 30 to 300 mL/min/100 mL (plasma flow ${F}_{P}$), 6–20 s (plasma mean transit time ${T}_{p}$), 40 to 60 mL/min/100 mL (tubular flow ${F}_{T}$) and 90 to 300 s (tubular mean transit time ${T}_{T}$). A literature based ${c}_{a}(t)$ was used [39], pre-padded with zeroes to create a 15 s baseline. Data were generated with Eq. (3) and $S_{0} \left( \user2{x} \right) = 1$ at pseudo-continuous temporal resolution 0.1 s, and then down-sampled to 1.1 s.

Three different types of motion were applied: no motion, rigid motion through sinusoidal vertical shifts with amplitude 12 pixels and period 4 s, and non-rigid motion derived from the deformation fields measured on a 2D clinical data set. The motion amplitudes were scaled up compared to the clinical data to test the performance of the approach under challenging conditions. The no-motion case is included to present a best-case scenario for MDR. Gaussian noise was added to the signal with varying standard deviations (SD) to test the noise sensitivity. Noise levels are expressed in terms of the contrast-to-noise ratio (CNR) defined as max ${(c}_{a})$/SD.

A particular issue in the design of DRO’s for motion correction is the choice of the ground truth. A result where the motion is frozen in a position that is different from that of the “ground truth” parameter maps is not necessarily incorrect as long as the frozen position is a natural breathing state. The problem is addressed in the DRO by applying the deformation fields to the “ground truth” parameter maps as well, and using as actual ground truth for error quantification the breathing state that minimises the difference between the reconstructed plasma flow and moving plasma flow map.

#### 2D clinical data

2D MRR data were taken from five consecutive subjects enrolled in an ongoing study into MRI biomarkers of renal fibrosis. The study was approved by the local research ethics committee, and all subjects gave written informed consent (Newcastle and North Tyneside 1 Research Ethics Committee, REC reference 14/NE/1120). MRR was performed in free-breathing using a 3.0 T scanner (Philips Achieva, Best, The Netherlands) and a 2D saturation-recovery turbo-flash sequence with linear encoding of *k*-space. A 2-channel body transmit coil was employed for homogeneous signal transmission and data were acquired using an 18-channel torso receive coil. Four slices (3 coronal, 1 axial) were acquired at a temporal resolution of 1.1 s. Other imaging parameters were as follows: acquisition matrix 116 (phase) × 135 (read), number of dynamics 250, echo time 1.63 ms, repetition time 3.6 ms, bandwidth 900 Hz, saturation recovery time 148 ms, flip angle 12^◦^, slice thickness 7 mm, sensitivity encoding (SENSE) factor 2.4, field of view 375 × 440 mm^2^, in-plane resolution 3.2 × 3.2 mm^2^, reconstructed matrix 480 × 480. The contrast agent Gd-DOTA (Dotarem, Guerbet, France) was injected with a half dosage of 0.05 mmol/kg body weight. The input ${c}_{a}(t)$ was measured in the aorta on the axial slice.

#### 3D clinical data

3D MRR data were taken from eight consecutive cases from a study on atherosclerotic renovascular disease [40]. The study was approved by the local research ethics committee, and all subjects gave written informed consent (Oldham Local Research Ethics Committee, REC reference 07/Q1405/21). MRR was performed using a 3.0 T whole body scanner (Philips Achieva, Philips Medical Systems) with a phased-array body coil for signal reception. Subjects were imaged using 3D spoiled gradient echo sequence in the oblique coronal plane. The following parameters were used for the acquisition: repetition time 5.0 ms, echo time 0.9 ms, field of view 400 × 400 × 100 mm, voxel size 3.13 × 3.13 × 4.0 mm^3^, flip angle 17^◦^, SENSE factor 2, acquisition matrix 128 × 84 × 10, reconstructed matrix 128 × 128 × 20. This resulted in a temporal resolution of 2.1 s/volume. Subjects were given a quarter dose of 0.025 mmol/kg GdDOTA (Dotarem, Guerbet, France) at a rate of 3 ml/s. In all cases, the acquisition and contrast agent injection were initiated simultaneously. The input ${c}_{a}(t)$ was measured in the aorta between the bifurcations of renal and iliac arteries.

### Assessment of registration

Registration quality in the DRO was measured by the percent error in any given field $P\left( {\mathbf{x}} \right)$, quantifying the difference between the reconstructed $P_{r} \left( \user2{x} \right)$ and the ground truth $P\left( \user2{x} \right)$:

19 $$E_{P} \left( \user2{x} \right) = \frac{{P_{r} \left( \user2{x} \right) - P\left( \user2{x} \right)}}{{\mathop {\max }\limits_{\user2{x}} \left\{ {P\left( \user2{x} \right)} \right\}}} \times 100$$

Bias of the result was quantified as the median $E_{P} \left( \user2{x} \right)$ across all ***x***, and the precision as the width of the 90% confidence interval. In addition to the quantitative metrics, registration quality was also assessed visually by comparing reconstructed parameter maps against exact maps $P\left( \user2{x} \right)$.

The registration quality in the patient data was assessed qualitatively by visual comparison of corrected and uncorrected parameter maps, time curves and model fits in motion-sensitive areas, and time-cut images.

Quantitative assessment of MDR was performed based on manual regions of interest (ROI) corresponding to the left kidney and right kidney. To create the ground truth, the left and right kidney were manually segmented on a single slice of 2D renal data. This was then propagated across all the time frames. The segmentation of the unregistered data was obtained by manually adjusting the position of the ROIs in every time frame to best follow the feature of interest. The same procedure applied to that of unregistered data was followed to obtain segmentation of registered data. Each segmentation was evaluated against the ground truth by calculating the Hausdorff distance using the *EvaluateSegmentation* tool [41]. A 2D slice was extracted from the middle of the 3D renal data and the same procedure applied to that of 2D renal data was followed to obtain the segmentations of 3D data.

To assess the impact of model bias, MDR was also applied with a simplified 3-parameter modified Tofts kinetic model [42]. The model is known to provide a poor fit to the data in the first pass, which introduces bias in the motion correction.

### Statistical analysis

The statistical analysis was performed using RStudio (version 4.0.3, 2020). Normality was assessed with Shapiro–Wilk test. Paired t test was performed to compare the Hausdorff distance between the unregistered and registered data. The 2D and 3D data were grouped together, and the analysis was carried out on the combined data. The significance level was set at 5%.

## Results

**Fig. 1:**
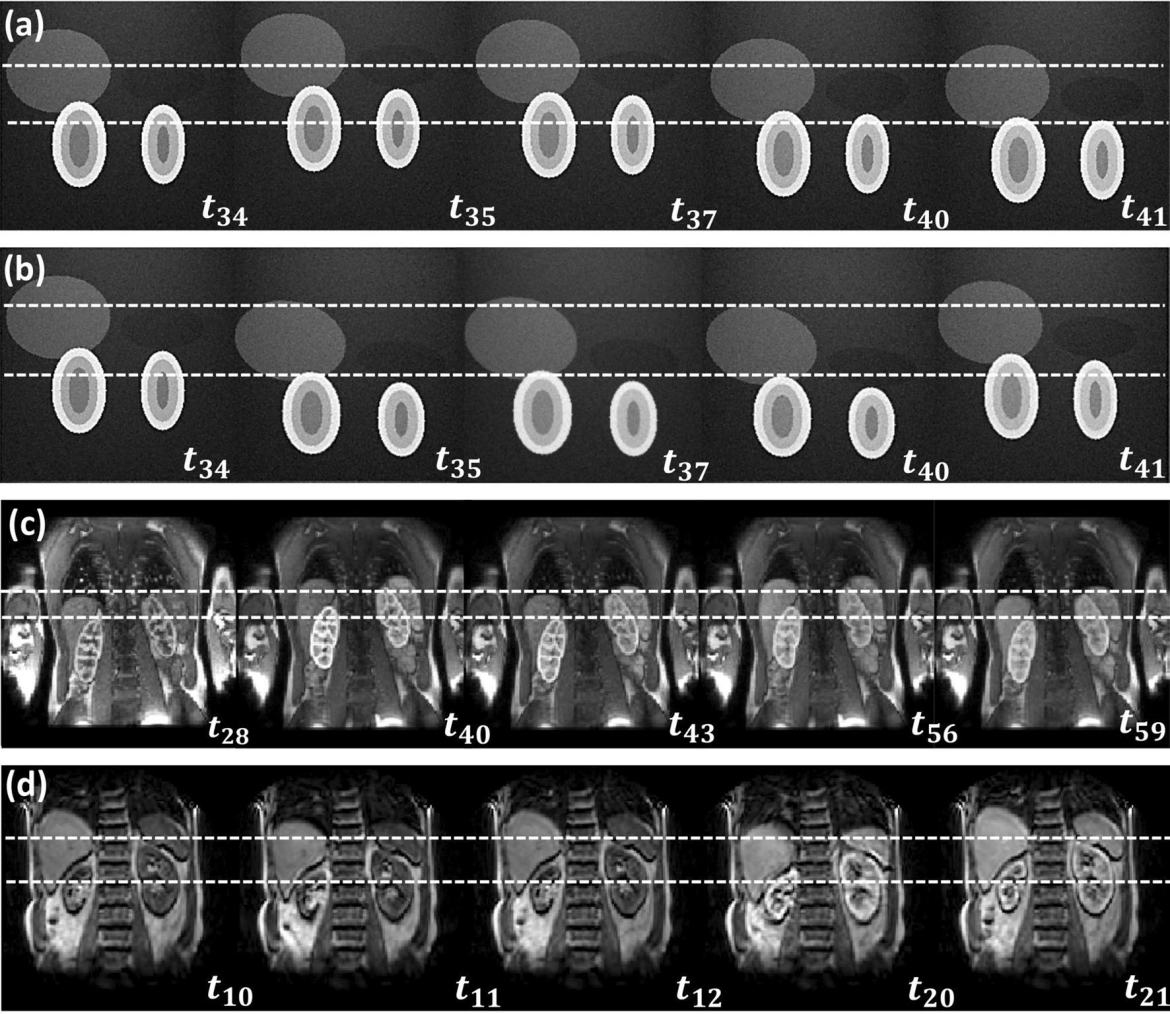
Original time series before motion correction with frame indices: **a** DRO data with rigid motion; **b** DRO data with non-rigid motion; **c** 2D MRR data for subject 3; **d** 3D MRR data for subject 1

Figure 1 illustrates the source data used in the study. The 3D data showed significant intra-frame artefacts, presenting a particular challenge for motion-correction.

**Fig. 2:**
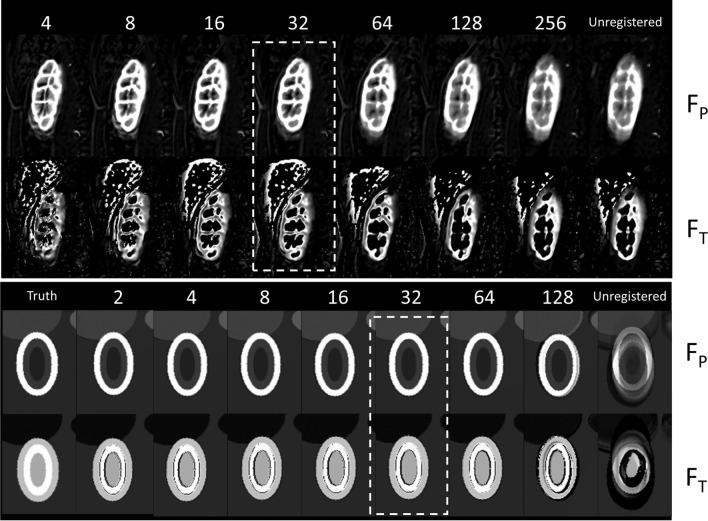
Selection of an optimal grid size ∆_*min*_ for the example of 2D patient data (top rows) and DRO with non-rigid motion (bottom rows). All calculations are performed at the lowest tolerance of *δ*_*min*_ = 0.1 pixels. For each dataset the figure shows the results for plasma flow (*F*_*P*_) and tubular flow (*F*_*T*_) at different grid sizes from lowest (left, 4 pixels and 2 pixels, respectively) to highest (right, 256 pixels and 128 pixels, respectively). The unregistered result is shown in the right most column. For the DRO the ground truth is given on the left. Motion corrections are performed over the entire image, but results are cropped to the right kidney to show the detail. The dashed lines show the selected grid size for each case. Dynamics, results for the other two parameters and error maps for the DRO are shown in the supplementary material

Figure 2 shows the effect of varying the deformation field gridsize ∆_min_ on the parameter maps. For the patient data, the ${F}_{P}$ maps show a gradual sharpening of the image until the smallest gridsize, but ${F}_{T}$ maps show that at the smallest gridsizes of 4- and 8-pixel small-scale deformations are induced that degrade the expected anatomical structure. Inspection of the dynamics suggests that at this stage, the deformation field attempts to correct for model errors. The optimal grid size for these data was chosen to 32 pixels (dashed lines) as no obvious further improvements are seen at 16. For the DRO, results improve when the grid size is reduced to 32 but no further improvements are seen beyond that. The effect of model bias does not play a role in the DRO because forward and inverse models were the same.

**Fig. 3:**
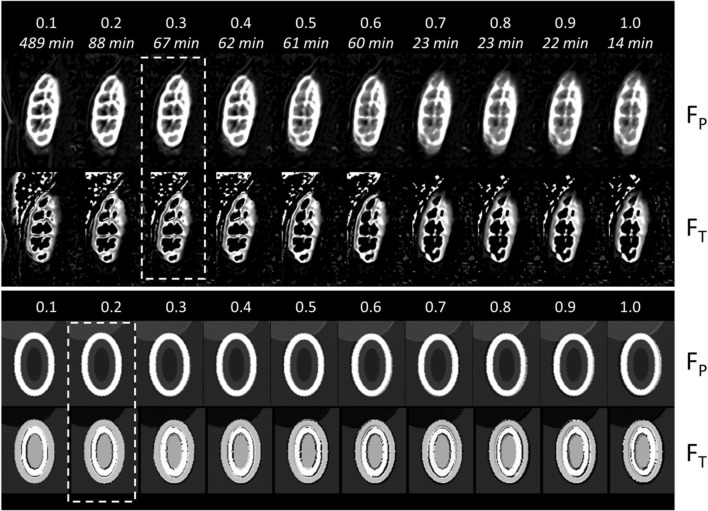
Selection of an optimal tolerance *δ*_min_ for the example of 2D patient data (top rows) and DRO with non-rigid motion (bottom rows). All calculations are performed at the optimal grid size of 32 pixels (see Fig. 2). For each dataset, the figure shows the results for plasma flow (*F*_*P*_) and tubular flow (*F*_*T*_) at different tolerances from lowest (left, 0.1 pixels) to highest (right, 1 pixel). For the patient data, the calculation times for each tolerance level are also shown. Motion corrections are performed over the entire image, but results are cropped to the right kidney to show the detail. The dashed lines show the selected tolerance for each case. Dynamics, results for the other two parameters and error maps for the DRO are shown in the supplementary material

Figure 3 shows the effect of varying the tolerance *δ*_min_. The patient data show that the results gradually converge to the result at the lowest tolerance of 0.1, at the cost of significant extra calculation time. With tolerances lower than 0.3, the calculation time increases dramatically from 67 min at 0.3 pixels to 88 min at 0.2 pixels and 489 min at 0.1 pixels. As there is no obvious improvement visible at tolerances lower than 0.3, this was chosen to be the optimal tolerance for these data—saving around 6 h of computation time per slice compared to the lowest tolerance of 0.1. The DRO shows a similar trend, but due to the sharp tissue interfaces, improvements continue to be apparent until a tolerance of 0.2 pixels.

**Fig. 4:**
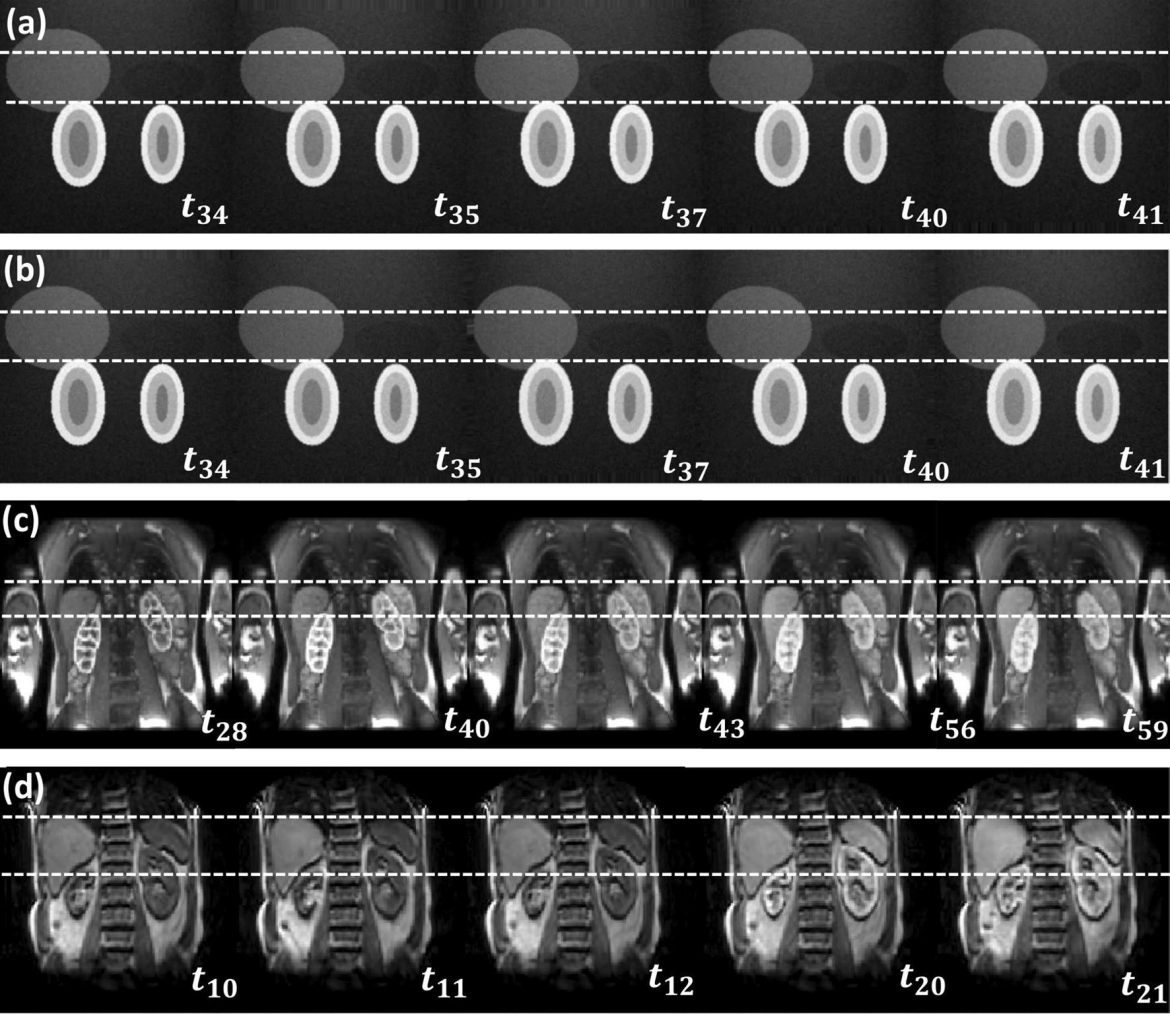
Illustration of the effect of motion correction at different time points with layout exactly as in Fig. 1. Time series after motion correction with frame indices: **a** DRO data with rigid motion; **b** DRO data with non-rigid motion; **c** 2D MRR data of subject 3; **d** 3D MRR data of subject 1

Figure 4 illustrates the results of MDR on the simulated data and a single clinical example. Comparison to the uncorrected data in Fig. 1 shows that the algorithm removes the motion effectively, including the exaggerated motion in the DRO.

**Fig. 5:**
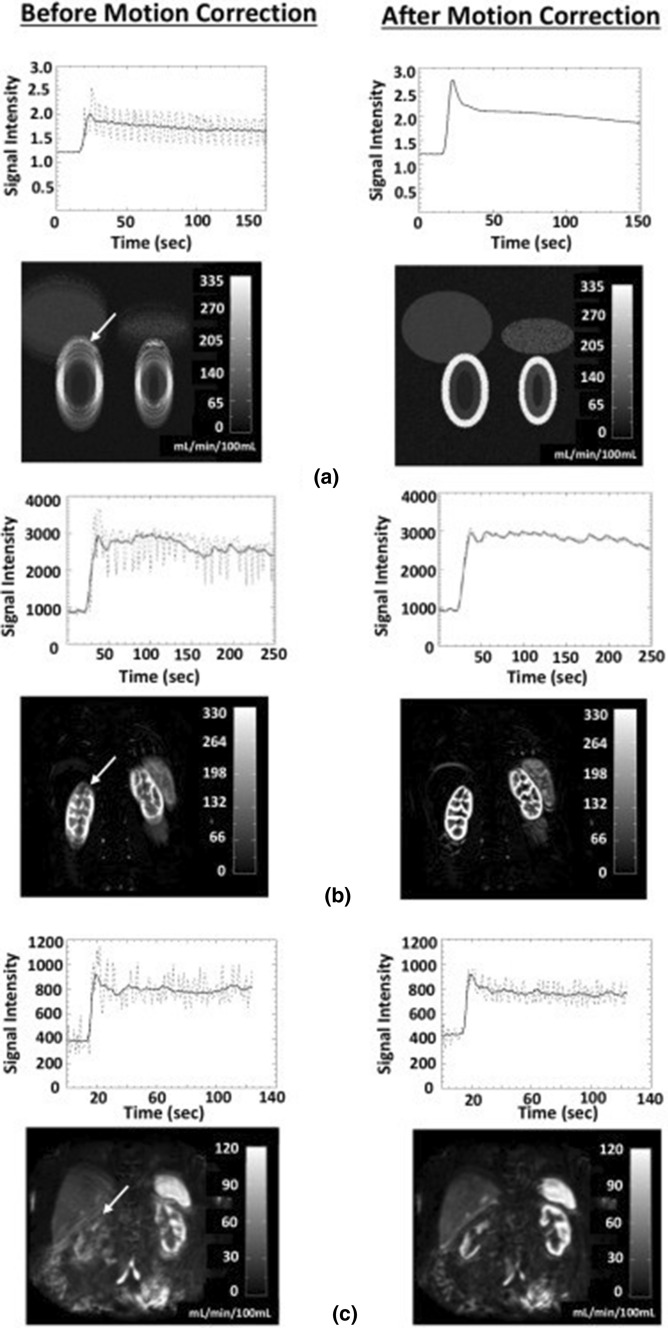
Effect of registration in (**a**) simulated data, (**b**) Effect of registration in (**a**) DRO data, (**b**) 2D MRR of subject 3 and **(c)** 3D MRR of subject 1. Arrows indicate the location of the ROI. The plots show the signal–time curves (dashed line) and the model fit (solid line) before and after motion correction. The plasma flow (*F*_*P*_) maps before and after motion correction are also presented

Figure 5 illustrates the effect of motion correction on temporal profiles, model fits and parameter maps for simulated and patient data. In all cases, the time curves show strong breathing-induced oscillations that are substantially reduced after motion correction, leading to a much-improved fit of the model to the data. In simulated and 2D data, the model describes the motion-corrected data almost exactly. In the 3D data, some oscillations remain after co-registration, consistent with the effects of within-frame motion artefacts. On the parameter maps, registration significantly reduces the motion-induced blurring that is visible on the uncorrected maps. This leads to clearer organ boundaries and delineation of internal anatomical structures, such as renal cortex and medulla.

**Fig. 6:**
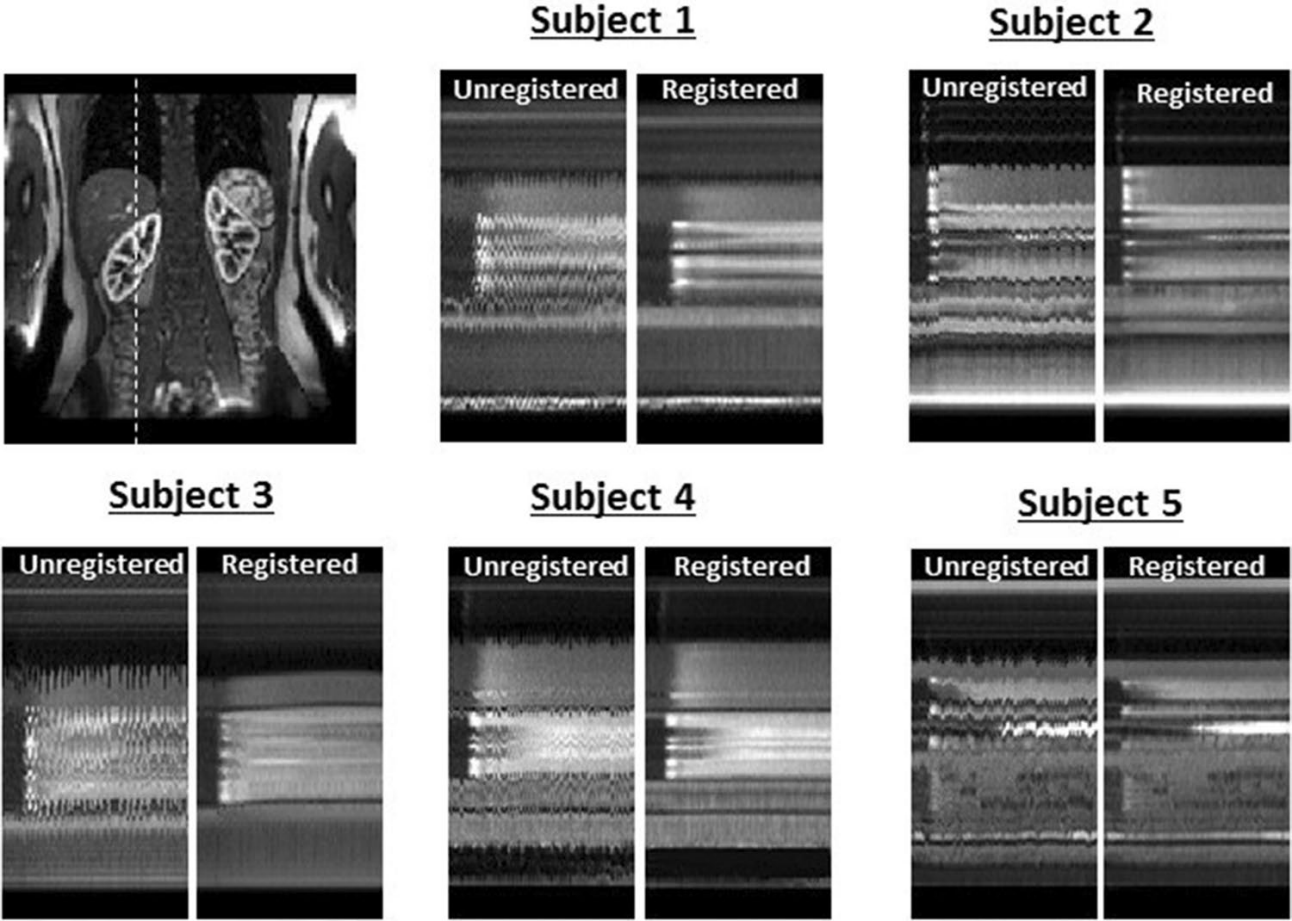
Effects of registration in superior–inferior direction in all 2D data sets. A coronal view is presented for anatomical reference, with a dashed line to indicate the location of the time cuts

Figure 6 shows the effect of registration through time cuts in all five 2D datasets. The time cuts of the unregistered data clearly show that significant motion is present which varies in structure and amplitude between subjects. The registered data demonstrate that the motion has effectively been removed, without affecting the changes in contrast. Typical calculation times for a single 2D slice were around 40 min on a laptop PC.

**Fig. 7:**
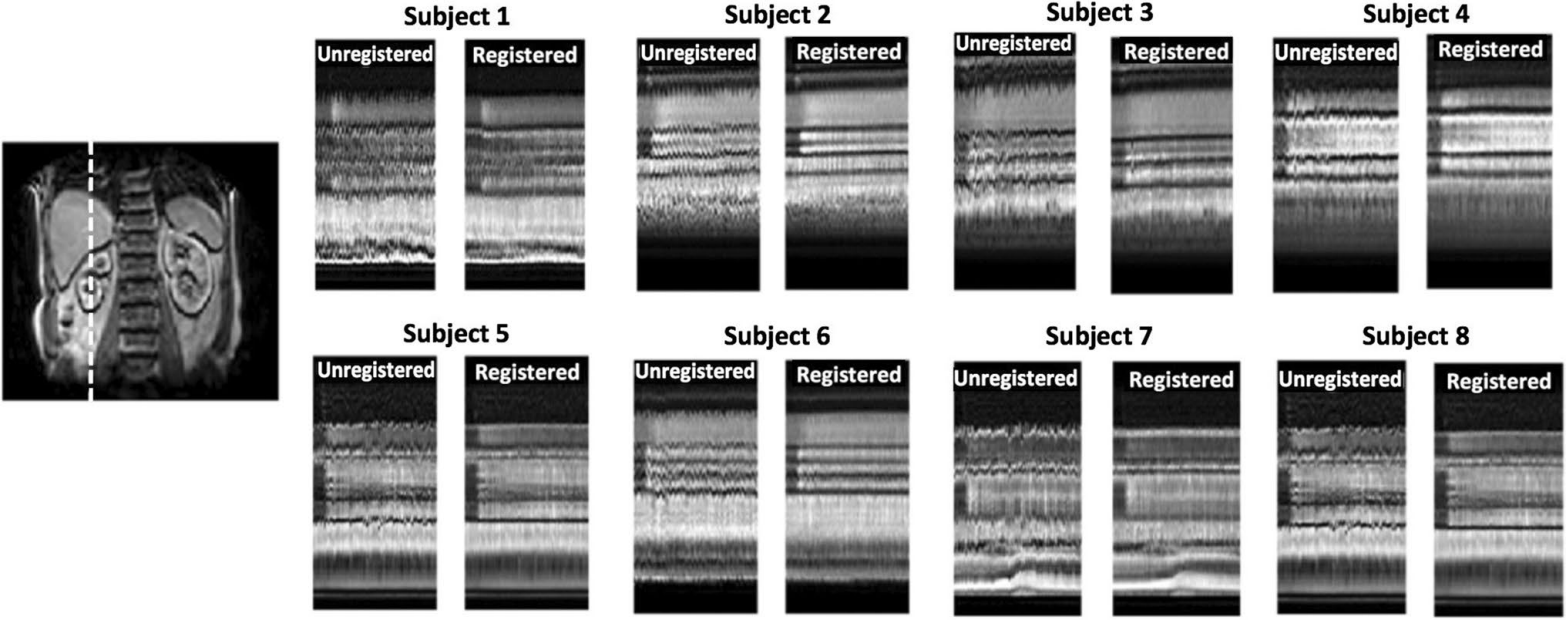
Effects of registration in superior–inferior direction in all 3D data sets. A coronal view is included for anatomical reference, with a dashed line to indicate the location of the time cuts

**Fig. 8:**
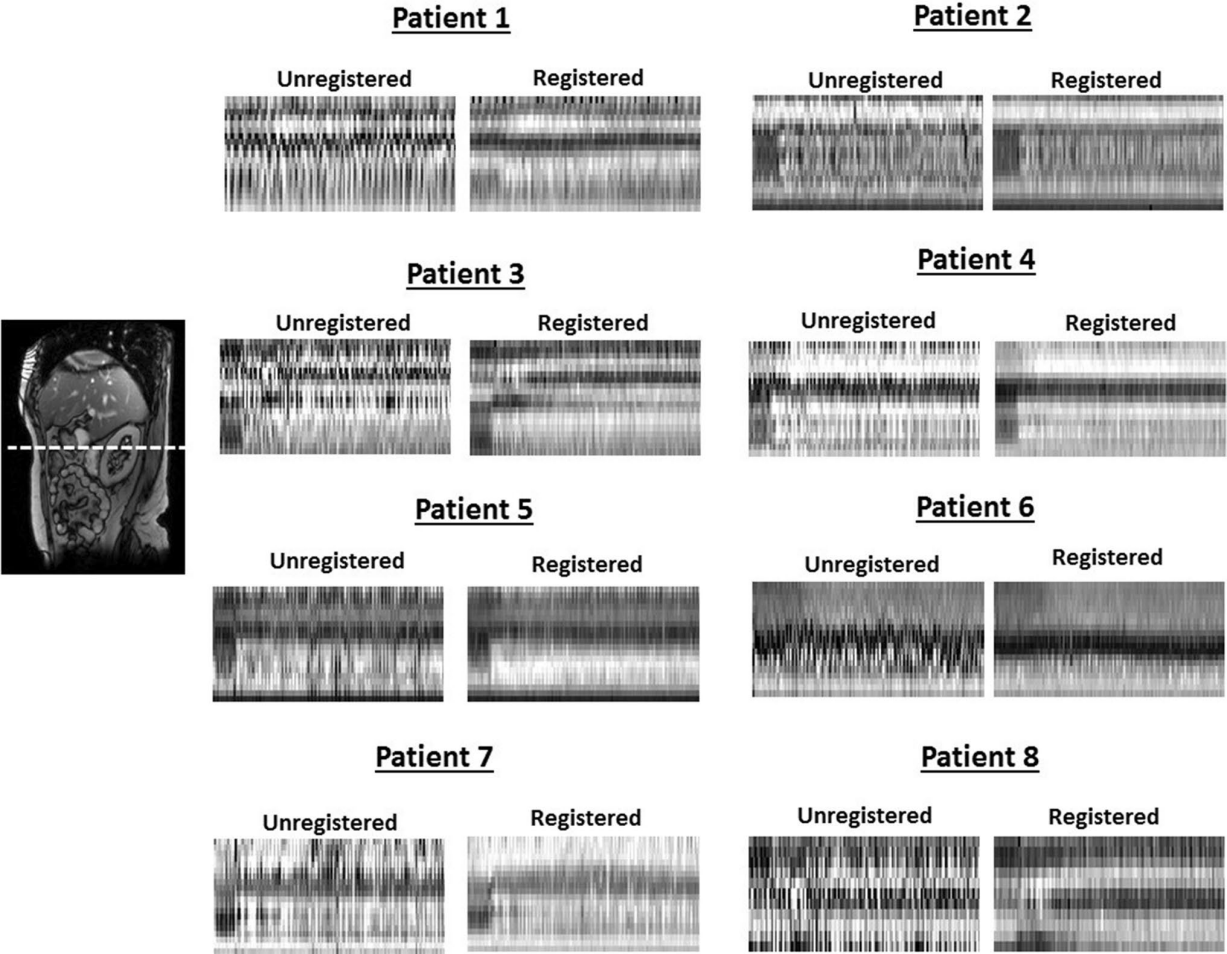
Effects of registration in anterior–posterior direction in all 3D data sets. A sagittal view is included for anatomical reference, with a dashed line to indicate the location of the time cuts

Figures 7 and 8 show the effect of registration through time cuts in all eight 3D datasets. Motion correction in the 3D data is more challenging due to the appearance of significant within-frame motion artefacts, which cannot be removed by image co-registration. Nevertheless, the time cuts clearly indicate effective reduction of motion effects. Visual inspection of the dynamic data demonstrates that kidney motion is effectively frozen, and any residual breathing-related oscillations are due to the within-frame artefacts. For all 3D data, the grid size was set to 4 pixels and tolerance to 0.2, leading to a calculation time of about 14 h on a laptop PC.

**Fig. 9:**
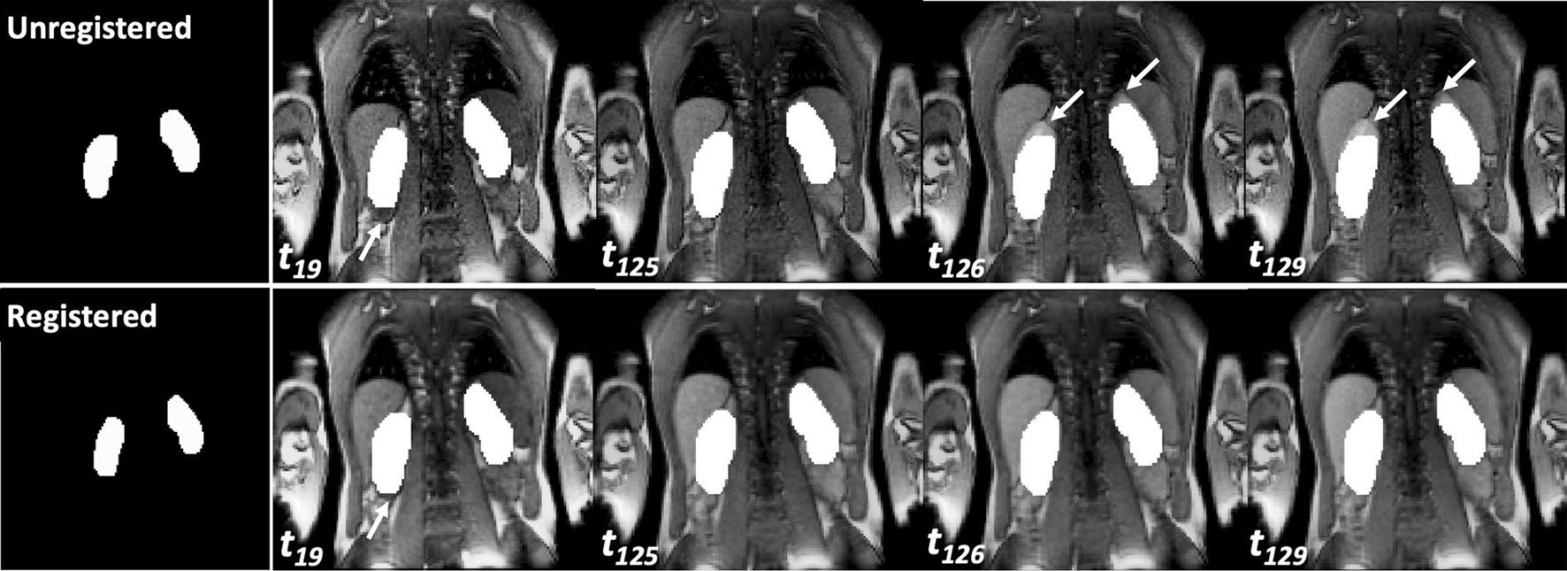
Presentation of images of 2D MRR data of subject 3. Upper row from left to right: A 2D manual segmentation of the left and right kidney. Time series before motion correction with frame indices and the overlay of the ground truth kidney segmentation. Arrows indicate areas where the ground truth segmentation mask does not align with the kidneys. Lower row from left to right: A 2D manual segmentation of the left and right kidney. Time series after motion correction with frame indices and the overlay of the ground truth kidney segmentation

Figure 9 shows the effect of registration through binary segmentation masks. Visual inspection of the ground truth segmentation mask demonstrates that kidney motion is efficiently removed. Table 1 presents the Hausdorff distance values used to assess segmentation quality before and after motion correction. Analysis of the 13 patients showed that the Hausdorff distance (HD) was significantly lower in segmentations after motion correction compared to segmentation masks before motion correction (HD_unregistered_ = 9.98 ± 9.76, HD_registered_ = 1.63 ± 0.49, *P* value = 0.00027).

**Table 1:** Quantitative performance of MDR algorithm on 2D and 3D clinical data

Image dimension	Subject	Hausdorff distance (voxels)	
		Unregistered	Registered
2D (*n* = 5)	Subject 1	12.04	1.41
	Subject 2	3.01	1.00
	Subject 3	31.76	2.23
	Subject 4	5.39	1.00
	Subject 5	3.61	1.00
3D (*n* = 8)	Subject 1	7.01	2.00
	Subject 2	5.83	1.96
	Subject 3	4.32	1.31
	Subject 4	4.47	2.23
	Subject 5	11.70	2.00
	Subject 6	30.36	1.41
	Subject 7	5.10	1.41
	Subject 8	5.09	1.41
2D/3D (*n* = 13)	*P* value	Mean ± sd	Mean ± sd
	0.00027	9.98 ± 9.76	1.63 ± 0.49

Numerical results present the Hausdorff distance (in voxels) between the data and ground truth mask

**Fig. 10:**
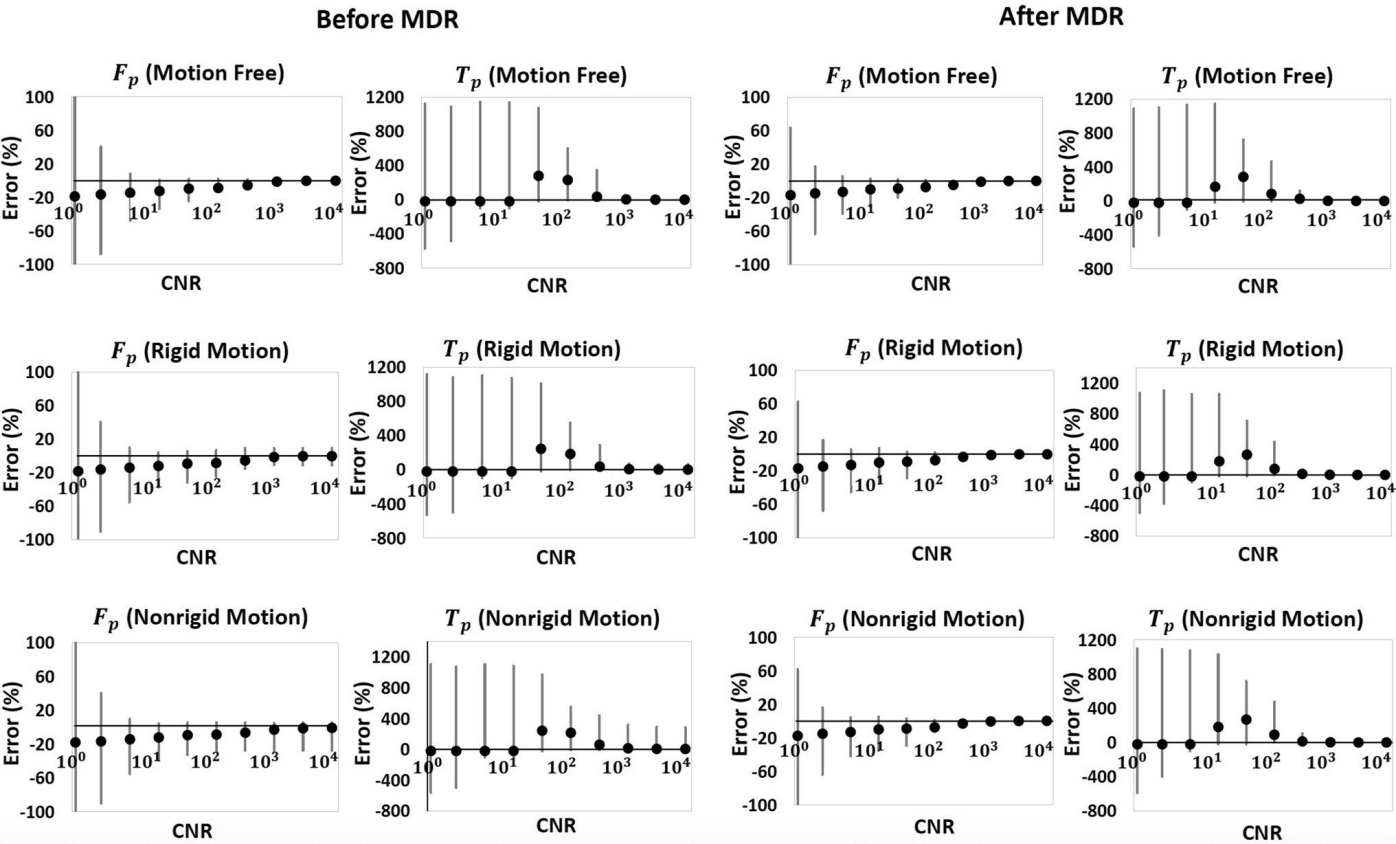
Error distribution before and after motion correction for the simulated data at CNR from 10^0^ (high noise level) to 10^4^ (low noise level). The columns show the two parameters and the rows show different motion types: no motion (top row), rigid motion (middle row), non-rigid motion (bottom row). The circles indicate the median relative parameter error for all the pixels in the image, and the error bars represent the 90% confidence intervals. For reasons of clarity, only two of the parameters are displayed; the trends in the other two parameters were similar

Figure 10 shows the error distribution as measured on the DRO before and after MDR, with CNRs ranging from high to low noise levels. The results in the motion-free case show that MDR improves the precision (width of 90% CI) at high and medium noise levels due to the spatial smoothing effect of the coordinate transformation. Comparing the results without MDR in the presence of rigid and non-rigid motion to the motion-free case, the figure shows that the motion induces additional error—mainly in the form of reduced precision under low noise conditions. At high noise conditions, the error is fully dominated by noise with no obvious added contribution of motion. After MDR, the error in data with rigid and non-rigid motion is reduced and becomes indistinguishable from that measured in motion-free data.

**Fig. 11:**
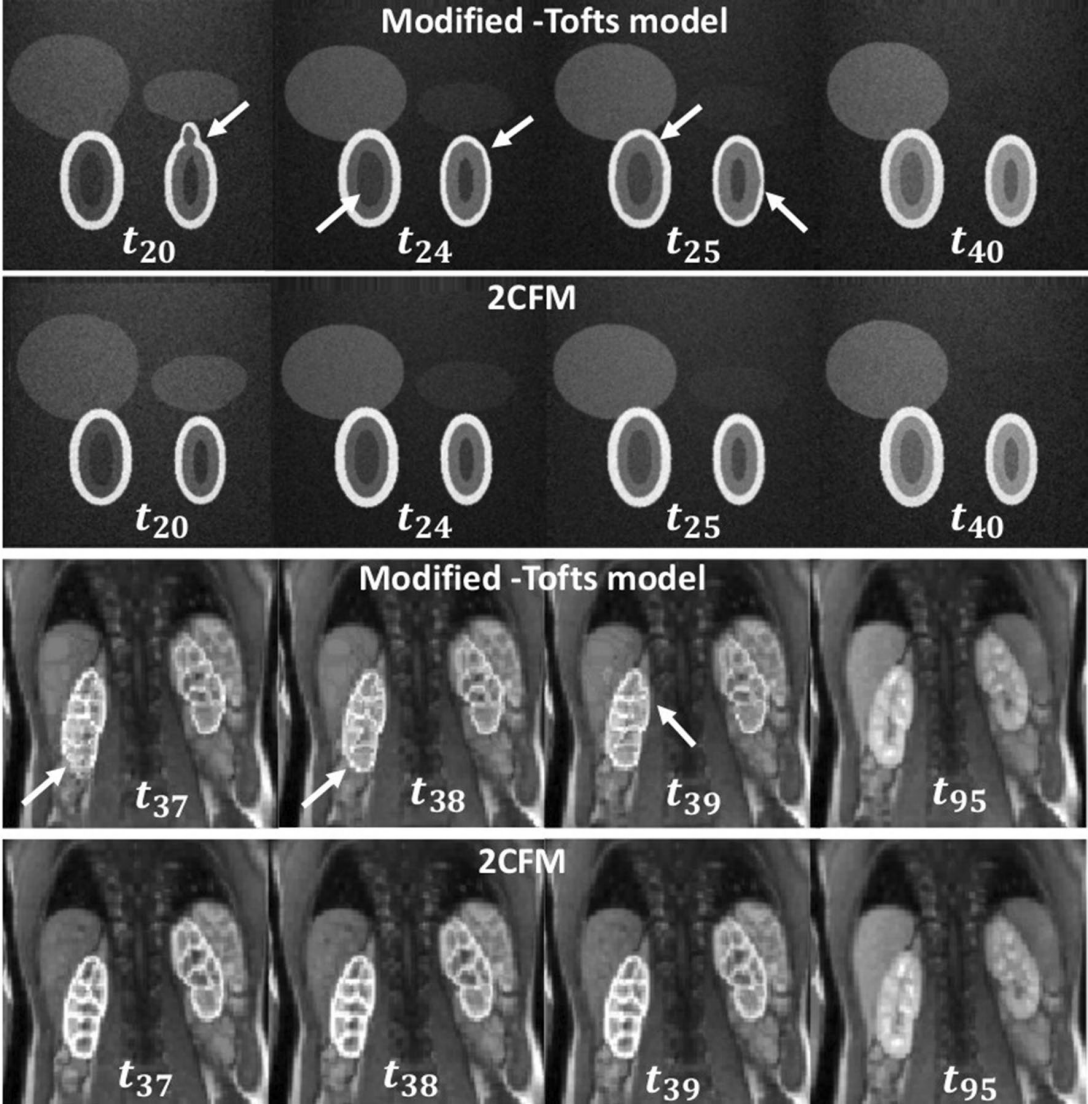
Illustration of the effect of motion correction at different time points using the modified Tofts model (top rows) and the two-compartment filtration model (2CFM) (bottom rows) for simulated data and patient data, respectively. Arrows indicate areas of poor registration due to model error

Figure 11 illustrates the effect of bias in the signal model. The modified Tofts model assumes infinite flow and therefore provides a poor fit to the earlier time points during the first pass of the contrast agent. As a result, the target for co-registration is a poor match to the actual image in the first pass, resulting in co-registration errors. This is illustrated in the figure: at the times corresponding to the first pass ($t_{{20}} ,~t_{{24}} ,~t_{{25}}$ in the simulated data and $t_{{37}} - t_{{39}}$ in patient data), the co-registered images are deformed.

## Discussion

The aim of this study was to investigate if MDR was suitable for motion correction in the challenging application area of free-breathing MRR at high temporal resolution. A key difference is that previous MDR methods [19, 23, 25, 28, 29] use pre-packaged co-registration modules, which does not allow to exploit the inherent symmetries of the problem to eliminate duplicate calculation steps. The MDR implementation proposed in this study uses a tailored FFD-based registration model which enables significant acceleration, allowing a co-registration in 3D with acceptable computation times on a standard PC without dedicated CPU implementations. Results in synthetic and patient data have shown that MDR effectively removes the motion in calculation times that are feasible on standard laptop computers.

MDR suffers from bias in cases where the chosen signal model does not adequately describe the data. Our results have confirmed this effect, showing significant unphysical deformations when an oversimplified model is used. The motion correction, when performed at small enough grid spacing, will in those conditions attempt to correct for errors in the signal model by collapsing or deforming areas of the image where the signal model does not fit the data. In qualitative imaging applications, MDR may therefore not be a preferred approach to motion correction [18]. However, in quantitative imaging a model-error will always bias the results, and this problem is not resolved by eliminating it from the motion-correction process. In fact, as our results show, the ensuing unphysical deformations help to flag up the presence of model error, where this may not be immediately obvious from a goodness-of-fit.

In a recent study, Coll-Font et al. [43] argued that MDR is unsuitable in the context of MRR because the signal models are valid only for the kidney, and that this therefore necessitates a region-of-interest-based co-registration. The predicted issues were not encountered in this study despite the approach of co-registering over the entire field of view. Indeed, what matters in MDR is the fit of the model to the data, and from this perspective, the renal compartmental model is no different than DCE-MRI models for other tissue types in the abdomen. The impulse response function is modelled as a bi-exponential, and it is only the interpretation of the model parameters that is specific to the kidney (Eq. 2). It should be noted though that this may not be true in other application areas. For instance, in cardiac DCE-MRI where an input function is taken in the left ventricle, the model will not be able to fit the lungs, pulmonary arteries or right ventricle as the bolus arrives in those tissues before the left ventricle. In this case, the validity of the model may indeed be more limiting, and the application may benefit from a model-free registration followed by a region-of-interest-based model fitting. An alternative solution though may be the use of MDR but with an input function selected in the pulmonary artery.

We used a FFD model for the breathing motion, as this is very generally applicable, can be easily adapted to motion at different scales by changing the smallest grid spacing, and is comparatively efficient computationally. However, other motion models may be used and could offer a benefit. For instance, a number of recent studies using MDR applied diffeomorphic transformations [30, 32], which are also free-form but derive deformations from stationary velocity fields. This ensures that the ensuing deformations are invertible, which is a necessary condition of any physical deformations. Diffeomorphic registrations will help to avoid unphysical deformations, producing more physical results even in the presence of model bias. This may be suitable in scenarios where an accurate physical model is not available or would be underdetermined by the available data.

In this study, we chose to use a newly built implementation of MDR without using pre-packaged solutions. For prototyping, this has the advantage of transparency, allowing a dissection of the different components, identification and precomputation of repeated calculation steps, and easy evaluation of alternative algorithm architectures. However, this also implies there is significant scope for acceleration using well-known recipes, such as stochastic gradients, preconditioner estimations for the line search [44], or optimizing at reduced resolution. Co-registrations at different times can be parallellized, and well-optimized CPU-type implementations of FFD can be used [45]. More generic registration packages, such as Elastix [46] and ANTs, [47] can easily be integrated to evaluate alternative approaches. Machine learning may also lead to step changes in computation times [48] and can presumably be implemented using unsupervised neural networks [49].

A separate potential route for improvement could involve a groupwise approach with the exact Eq. (16) rather than the approximation $\left( {\partial \user2{R}} \right)_{{ts}} \left( {\user2{x},s} \right) = ~\delta _{{ts}}$. This was explored in preliminary experiments and abandoned after these demonstrated extremely slow convergence without obvious benefit. Nevertheless, the approach has not been evaluated systematically and can potentially be improved using strategies, such as joint optimization of deformation fields and parameter maps. The alternative approach of using the analytical model solution (Eq. 3), which fulfills the condition $\left( {\partial \user2{R}} \right)_{{ts}} \left( {{\mathbf{x}},s} \right) = ~\delta _{{ts}}$, was also explored. The approach was abandoned after preliminary experiments demonstrated salt-and-pepper noise that dominated the cost function in the co-registration step. However, potential solutions, such as improved non-linear optimization or de-noising, have not been explored.

While the focus of this study is on retrospective motion correction by image registration, this is not the only means of correcting or reducing motion in medical imaging. Other approaches include motion-corrected compressed sensing [50, 51], prospective or retrospective gating, breath holding, using k-space trajectories that are less motion-sensitive [52], or a combination of the above [17]. These methods can be combined with MDR, and there may also be a benefit in integrating MDR with image reconstruction to produce a joint model-driven reconstruction [53, 54] and motion correction. Finally, while we have demonstrated that MDR effectively corrects for breathing motion in MRR, future studies are needed to determine whether MDR improves on alternative model-free approaches that have been proposed in this context [15, 43].

## Conclusion

MDR provides effective motion correction in the challenging application of free-breathing MRR at high temporal resolution. Future studies are needed to determine whether MDR improves the results compared to alternative co-registration methods proposed in this context.

## Appendix

We provide more detailed proof of Eq. (16) to calculate the partial derivative of the cost function with respect to $\user2{D}_{j} \left( k \right)$. First, we show that the partial derivative of the deformed signal is given by:

20 $$\frac{{\partial S_{\user2{D}} \left( {\user2{x},t} \right)}}{{\partial \user2{D}_{j} \left( t \right){\text{~}}}} = ~W_{j}^{{{\Delta }}} \left( \user2{x} \right)\left( {\nabla S} \right)\left( {\user2{D}\left( {\user2{x},t} \right),t} \right)~$$

This can be proven using Eq. (10). Consider for instance the derivative with respect to the *x*-component $\user2{D}_{{x,j}} \left( t \right)$ of $\user2{D}_{j} \left( t \right)$, using Eq. (7) for $\user2{D}\left( {\user2{x},t} \right)$:

$$\begin{aligned} \frac{{\partial S_{\user2{D}} \left( {\user2{x},t} \right)}}{{\partial D_{{x,j}} \left( t \right){\text{~}}}} = ~ & \mathop \sum \limits_{l} \frac{{\partial W_{l}^{\delta } }}{{\partial x}}\left( {\user2{D}\left( {\user2{x},t} \right)} \right)\frac{{\partial D_{x} \left( {\user2{x},t} \right)}}{{\partial D_{{x,j}} \left( t \right)}}S_{l} \left( t \right) \\ ~ = \mathop \sum \limits_{l} \frac{{\partial W_{l}^{\delta } }}{{\partial x}}\left( {\user2{D}\left( {\user2{x},t} \right)} \right)W_{j}^{\Delta } \left( \user2{x} \right)S_{l} \left( t \right) \\ ~ = W_{j}^{\Delta } \left( \user2{x} \right)\mathop \sum \limits_{l} \frac{{\partial W_{l}^{\delta } }}{{\partial x}}\left( {\user2{D}\left( {\user2{x},t} \right)} \right)S_{l} \left( t \right) \\ = W_{j}^{\Delta } \left( \user2{x} \right)\frac{{\partial S}}{{\partial x}}\left( {\user2{D}\left( {\user2{x},t} \right),t} \right)~~~~ \\ \end{aligned}$$

Now we calculate the partial derivative of the cost function (Eq. 15):

$$\begin{aligned} \frac{{\partial \chi ^{2} }}{{\partial \user2{D}_{j} \left( t \right)}} = & ~\mathop \sum \limits_{s} \mathop \sum \limits_{{\mathbf{x}}} R\left( {\user2{x},s} \right)\frac{{\partial R\left( {\user2{x},s} \right)}}{{\partial \user2{D}_{j} \left( t \right)}} \\ ~ = \mathop \sum \limits_{s} \mathop \sum \limits_{{\text{x}}} R\left( {\user2{x},s} \right)\left( {\partial _{t} R} \right)\left( {\user2{x},s} \right)\frac{{\partial S_{D} \left( {\user2{x},t} \right)}}{{\partial \user2{D}_{j} \left( t \right)}} \\ = \mathop \sum \limits_{s} \mathop \sum \limits_{{\text{x}}} R\left( {\user2{x},s} \right)\left( {\partial _{t} R} \right)\left( {\user2{x},s} \right)W_{j}^{{{\Delta }}} \left( \user2{x} \right)\left( {\nabla S} \right)_{\user2{D}} \left( {\user2{x},t} \right) \\ ~ = \mathop \sum \limits_{{\text{x}}} W_{j}^{{{\Delta }}} \left( \user2{x} \right)\left( {\nabla S} \right)_{\user2{D}} \left( {\user2{x},t} \right)\mathop \sum \limits_{s} R\left( {\user2{x},s} \right)\left( {\partial _{t} R} \right)\left( {\user2{x},s} \right) \\ ~ = \mathop \sum \limits_{{\text{x}}} W_{j}^{{{\Delta }}} \left( \user2{x} \right)\left( {\nabla S} \right)_{\user2{D}} \left( {\user2{x},t} \right){~\mathcal{R}}\left( {\user2{x},t} \right) \\ \end{aligned}$$

We used Eq. (16) in the last line, which leads to Eq. (13) with the substitution $R \to {\mathcal{R}}$.

## Footnotes

1 We used the notation $S\left( {{\mathbf{x}}, - } \right),~$ to indicate that the right-hand side can depend on signal values $S\left( {{\mathbf{x}},t} \right),$ at any *t*.
